# Evolution of a Project to Improve Inpatient-to-Outpatient Dermatology Care Transitions: Mixed Methods Evaluation

**DOI:** 10.2196/43389

**Published:** 2023-05-25

**Authors:** Samantha M R Kling, Maria A Aleshin, Erika A Saliba-Gustafsson, Donn W Garvert, Cati G Brown-Johnson, Alexis Amano, Bernice Y Kwong, Ana Calugar, Jonathan G Shaw, Justin M Ko, Marcy Winget

**Affiliations:** 1 Division of Primary Care and Population Health Department of Medicine Stanford University School of Medicine Palo Alto, CA United States; 2 Department of Dermatology Stanford University School of Medicine Stanford, CA United States; 3 Ambulatory Quality Department Stanford Health Care Stanford, CA United States

**Keywords:** care transition, discharge planning, inpatient, outpatient, follow-up, teledermatology, dermatology, consultative dermatology

## Abstract

**Background:**

In-hospital dermatological care has shifted from dedicated dermatology wards to consultation services, and some consulted patients may require postdischarge follow-up in outpatient dermatology. Safe and timely care transitions from inpatient-to-outpatient specialty care are critical for patient health, but communication around these transitions can be disjointed, and workflows can be complex.

**Objective:**

In this 3-phase quality improvement effort, we developed and evaluated an intervention that leveraged an electronic health record (EHR) feature, known as SmartPhrase, to enable a new workflow to improve transitions from inpatient care to outpatient dermatology.

**Methods:**

Phase 1 (February-March 2021) included interviews with patients and process mapping with key stakeholders to identify gaps and inform an intervention: a SmartPhrase table and associated workflow to promote collection of patient information needed for scheduling follow-up and closed-loop communication between dermatology and scheduling teams. In phase 2 (April-May 2021), semistructured interviews—with dermatologists (n=5), dermatology residents (n=5), and schedulers (n=6)—identified pain points and refinements. In phase 3, the intervention was evaluated by triangulating data from these interviews with measured changes in scheduling efficiency, visit completion, and messaging volume preimplementation (January-February 2021) and postimplementation (April-May 2021).

**Results:**

Preintervention pain points included unclear workflow for care transitions, limited patient input in follow-up planning, multiple messaging channels (eg, EHR based, email, and phone messages), and time-inefficient patient tracking. The intervention addressed most pain points; interviewees reported the intervention was easy to adopt and improved scheduling efficiency, workload, and patient involvement. More visits were completed within the desired timeframe of 14 days after discharge during the postimplementation period (21/47, 45%) than the preimplementation period (28/41, 68%; *P*=.03). The messaging workload also decreased from 88 scheduling-related messages sent for 25 patients before implementation to 30 messages for 8 patients after implementation.

**Conclusions:**

Inpatient-to-outpatient specialty care transitions are complex and involve multiple stakeholders, thus requiring multifaceted solutions. With deliberate evaluation, broad stakeholder input, and iteration, we designed and implemented a successful solution using a standard EHR feature, SmartPhrase, integrated into a standardized workflow to improve the timeliness of posthospital specialty care and reduce workload.

## Introduction

Changes in health care financing, including adoption of diagnosis-related groups, have had a negative effect on coverage and reimbursement for inpatient dermatological care [1-3]. In response, inpatient dermatological care has shifted from dedicated dermatology wards to consultative services [1,3-5]. Thus, patients with dermatologic disorders are admitted to inpatient services attended by nondermatologist providers, and in-hospital dermatological care is provided by consulting dermatologists. Some consulted patients may require postdischarge follow-up dermatological care provided in outpatient clinics. Coordination of transitions requires close collaboration between multiple stakeholders, including the primary inpatient team, dermatology team (dermatologists and residents), clinic staff, and patients, to plan, schedule, and execute follow-up care [6].

Care transition workflows are complex with multiple stakeholders. Poor coordination has wide-ranging impacts from frustration to complications resulting in suboptimal health, excess cost, and hospital readmissions [2,7]. Timeliness of transitions is associated with higher follow-up visit attendance [8], but structural and organizational barriers and communication deficits contribute to gaps in coordination [9,10]. Shifting transitions from siloed, disease-centric care to integrated, patient-centered care [11,12] can improve health outcomes, readmissions, costs, and patient satisfaction [13]. Nurse-led transition support can help patients and caregivers navigate the health system but is resource intensive and may not be feasible in all situations [14]. Easy-to-use, rapidly deployable solutions are needed to support effective and timely care transitions from inpatient consultations to outpatient specialty care.

Widespread outpatient teledermatology use has allowed greater flexibility for scheduling dermatology patients [15-17]. However, concerns regarding communication, timely follow-up, and excessive workload remain among clinicians and staff, prompting this quality improvement (QI) project. In this study, we described our efforts to improve dermatology care transitions in three phases: (1) redefining the problem and solution development; (2) exploring preintervention pain points and adapting the solution; and (3) evaluating the intervention and identifying persisting challenges.

## Methods

### Study Setting and Patients

Stanford Medicine’s Dermatology Department (Bay Area, California) has 13 outpatient clinics with 16 subspecialties and provides 2 inpatient consultative services in a quaternary hospital: general dermatology and supportive dermato-oncology, a service for oncology patients with dermatological complications. A total of 5 dermatologists and rotating residents (2 per month) provide inpatient consultations to >1500 inpatients per year and outpatient follow-up care. Approximately 40% of patients who receive an inpatient dermatology consultation require postdischarge dermatology follow-up; many are affected by complex, high-risk skin conditions; are immunocompromised; and have multidisciplinary inpatient care teams. Care transitions also involved scheduling staff, including front office scheduling staff, new patient coordinators (NPCs), and their managers.

### QI Process, Data Collection, and Analysis

#### Phase 1: Redefining the Problem and Solution Development

##### Current State

**Figure 1:**
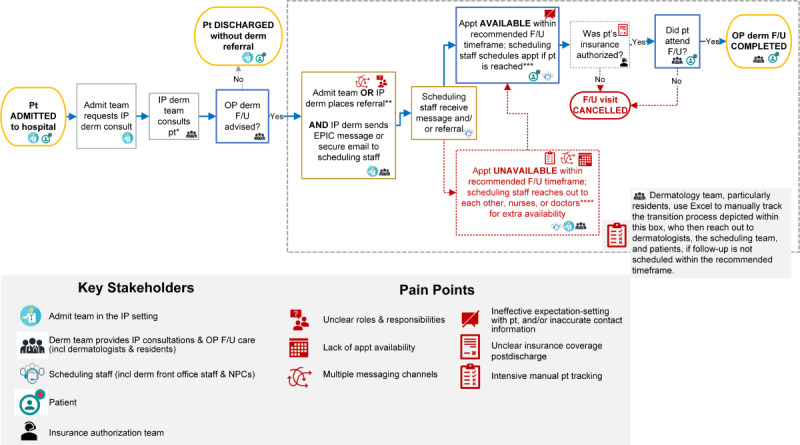
Preintervention (ie, baseline) process mapped using insights from schedulers, insurance authorization, dermatologists, and residents in phase 1 and redefining the problem and solution development of a quality improvement project aiming to improve timeliness of transitioning patients from inpatient-to-outpatient dermatology and reduce workload. *Inpatient dermatology consult completed in person or via e-consult. **Could occur pre or postdischarge. If before, scheduling team may wait to initiate scheduling until patient is discharged. ***Patient contacted by phone up to three times and sent a mailed letter. ****Dermatology team, particularly residents, manually track patient transitions in Excel who reached out to dermatologists, scheduling staff, and patients if follow-up not scheduled within the recommended timeframe. Admit: Admitting; Appt: Appointment; Derm: dermatology; F/U: follow-up; incl: including; IP: inpatient; NPC: new patient coordinator; OP: outpatient; pt: patient.

In February 2021, clinical leaders (MAA, JMK, BYK, and AC) met with scheduling staff and insurance authorization representatives to understand concerns regarding inpatient-to-outpatient care transitions. These insights were combined with informally collected anecdotal experiences of dermatologists and residents in the preintervention process map (Figure 1). In brief, the preintervention workflow had the following steps:

1. Admitting team (eg, general medicine) requested a dermatology consultation.
2. Inpatient dermatology team (dermatologists and residents) conducted a consultation with the patient and would determine if outpatient follow-up is needed.
3. The admitting team or dermatology team would place the referral. A member of the dermatology team would also contact the scheduling staff via electronic health record (EHR)–based message and a secure email or telephone call.
4. Scheduling staff would receive the message, identify the correct work queue, and contact the patient to schedule an appointment.
5. The dermatology team, primarily residents, would monitor the patients’ EHR to determine if patient was scheduled for follow-up, had insurance approval, and completed follow-up visit. Resident would contact the scheduling staff via EHR-based message and a secure email or telephone call if any step of the process fell through.

Key pain points included (1) multiple and inconsistent communication channels (eg, EHR-based messages, emails, and phone calls), (2) unclear roles and responsibilities, (3) burdensome workload for dermatology and scheduling teams, and (4) intensive manual tracking of the process to schedule follow-ups. These pain points may be exacerbated in the consultative context; the inpatient dermatology team consults for numerous services, such as medicine, surgery, intensive care, and obstetrics and gynecology, resulting in many workflows to navigate. Furthermore, skin issues will often not completely resolve by discharge, despite the dermatology consultative team following up with patients closely throughout their hospitalization. Thus, patients may benefit from outpatient skin-directed follow-up; enabling this care faces substantial barriers. In particular, patients’ dermatologic diagnoses may often be unrelated to their primary reason for hospitalization, which may result in a deprioritization (by both patients and providers) of skin issues after discharge.

##### Patient Perspectives

Phase 1 also included formative qualitative interviews conducted to understand patients’ and caregivers’ experiences with inpatient-to-outpatient dermatology care transition and elucidate their needs. A total of 14 patients and 1 caregiver were interviewed; methods and patient characteristics can be found in Multimedia Appendix 1.

Patients were satisfied with discharge plans and communication with their dermatologists; however, issues persisted around 3 themes: communication and expectation setting during discharge planning; dermatology team support and postdischarge teledermatology; and care coordination and prioritization for medically complex patients. Multimedia Appendix 2 contains supporting quotations. Notably, patients who experienced serious, nondermatological medical events had limited recall of interactions with dermatology. Consequently, for some patients, the necessity of dermatology follow-up was unclear, but caregivers could provide indispensable support. Prioritizing other health issues and coordinating dermatology care with other teams also impacted follow-up success. Nevertheless, patients who followed up with dermatology were generally satisfied with their follow-up coordination experience, even if delayed. They appreciated the dermatology team’s accessibility during transition, facilitated for some patients by teledermatology and direct provider messaging. Despite not all patients following up via video and some still preferring in-person visits, most agreed that video visits are convenient, easy, and provide better access. In fact, almost all patients expressed interest in future video visits, although concerns remained around photo or video quality.

##### Intervention Development

The developed intervention included a SmartPhrase (also known as a dot phrase), a flexible Epic EHR feature that creates templates (eg, fillable statement or table) that can be integrated into patient notes, referrals, or discharge instructions by typing a period and short phrase [18]. On the basis of the patient interviews and informal data collection with inpatient dermatologists, dermatology residents, NPCs, front office schedulers, nurses, and insurance representatives, a team drafted a SmartPhrase form that addressed the main pain points. In particular, the SmartPhrase addressed schedulers’ request for more information, including recommended follow-up timeline, preferred patient contact information, and preferred provider for scheduling and requests for overbooking. Including the reason for follow-up and follow-up timeline was particularly important for confirming patient’s understanding of the importance of follow-up. Patients’ perspectives prompted the inclusion of a field to indicate whether a video visit would be an acceptable option. To address the patients’ perspective that their dermatologic condition was not always their priority, the SmartPhrase was completed with the patient at the bedside to explain the importance of follow-up to the patient and caregiver. The team shared the SmartPhrase with residents and incorporated their feedback before integrating it into Epic.

The SmartPhrase (Table 1) prompted dermatologists to obtain and document pertinent patient information necessary for scheduling follow-up care in the new workflow:

1. Dermatology team (dermatologists and residents) engages in shared decision-making with patients and caregivers during an inpatient consultation.
2. Dermatology team obtains the necessary information to schedule follow-up and documents in the SmartPhrase during inpatient consultation.
3. Dermatology team submits an “as soon as possible (ASAP)” referral for outpatient care that includes completed SmartPhrase.
4. NPCs receive the referral. NPCs keep new patient referrals in their work queue and forward return patient referral to the front office scheduling staff.
5. Scheduling team, NPCs and front office scheduling staff, facilitate correct and timely scheduling of follow-up visits with patient.
6. Scheduling team connects with dermatology team about patients who decline follow-up or cannot be reached to determine the next steps.

The intervention, the SmartPhrase and associated workflow, was activated on March 22, 2021. Dermatologists (n=5), rotating residents (2 per month), and 15 scheduling staff received group verbal training with written documentation describing the workflow and SmartPhrase use from an improvement leader. A total of 13 scheduling staff, including front office scheduling staff and NPCs and their 2 managers, were also trained.

**Table 1:** Content of developed electronic health record–based SmartPhrase table, a key component of the associated workflow, that prompted predischarge conversations with the patient to capture and document necessary patient information to schedule follow-up for patients transitioning from inpatient-to-outpatient dermatology care. At the top of the SmartPhrase table, there were two notes that emphasized steps in the workflow: (1) “PLEASE MARK ALL INPATIENT REFERRALS AS PRIORITY: ASAP” and (2) “PLEASE PLACE THIS DOT PHRASE INTO THE COMMENTS SECTION OF THE REFERRAL.” The first statement was added after initial implementation and was based on early feedback from dermatologists, residents, and scheduling staff.

SmartPhrase component and generalizable drop-down menu options		Example of content within an ASAP^a^ referral for outpatient follow-up care
**Patient name on file**		
	[Patient name from patient’s electronic health record]	Jane Doe
**Date of birth on file**		
	[Patient date of birth from patient’s electronic health record]	00/00/0000
**Patient ID**		
	[Patient ID from patient’s electronic health record]	00000000
**REASON FOR REFERRAL: Patient recently admitted to hospital, seen by inpatient derm^b^ team, needs outpatient dermatology follow-up for: (Reason for derm DC^c^ referral: 45,992)**		
	SCAR^d^ (SJS^e^ or TEN^f^, DRESS^g^, AGEP^h^)	SSTI^i^
	Blistering dermatitis (PV^j^, BP^k^, Linear IgA^l^, etc)	—^m^
	GVHD^n^	—
	Chemo^o^ or immunotherapy-related rash	—
	Vasculitis	—
	Connective tissue disease (lupus, DM^p^, etc)	—
	Neutrophilic dermatosis (PG^q^, sweets^r^, etc)	—
	SSTI	—
	Skin exam	—
	Other^s^	—
**IS THIS A NEW OR RETURN PATIENT (New or return patient: 46914)**		
	New	Return
	Return	—
**TIME REQUESTED FOR FOLLOW-UP: (Time requested for derm appt: 46001)**		
	1-3 days	2 weeks
	1 week	—
	2 weeks	—
	1 month	—
	2 months	—
	Next available (nonurgent follow-up)	—
	Discharge clinic VV only (gen^t^ derm): Wednesday AM	—
	Discharge clinic CC^u^ only (SDO^v^)—Tuesday PM	—
	[Insert specific date]	—
**VISIT TYPE REQUESTED: (Visit type requested: 46002)**		
	No preference	Video visit
	In person	—
	Video visit	—
	E-consult	—
	Other^s^	—
**LOCATION PREFERRED: (Location of derm follow-up: 46003)**		
	[Drop-down list of outpatient dermatology clinics]	Clinic name
**PROVIDER PREFERRED: {Provider preferred for derm follow-up: 46006)**		
	[Drop-down list of dermatology providers]	Provider name
**INTERPRETER NEEDED FOR SCHEDULING AND VISIT: (Interpreter Needed: 46005)**		
	Yes	Yes
	No	—
**PREFERRED LANGUAGE SPOKEN: (Preferred Language: 46006)**		
	English	Spanish
	Spanish	—
	Mandarin	—
	Russian	—
	Vietnamese	—
	Other^s^	—
**BEST CONTACT NUMBER OR WHO TO CONTACT TO SCHEDULE: (Best contact: 46007)**		
	[Text box]	123-345-5678 James Doe (spouse)

^a^ASAP: as soon as possible.

^b^derm: dermatology.

^c^DC: discharge.

^d^SCAR: severe cutaneous adverse reactions.

^e^SJS: Stevens-Johnson syndrome.

^f^TEN: toxic epidermal necrolysis.

^g^DRESS: drug reaction with eosinophilia and systemic symptoms.

^h^AGEP: acute generalized exanthematous pustulosis.

^i^SSTI: skin soft tissue infection.

^j^PV: Pemphigus vulgaris.

^k^BP: bullous pemphigold.

^l^IgA: immunoglobin A.

^m^Clinicians were to select the relevant options from the SmartPhrase for each individual patient. For the provided example, 1 option was selected from each SmartPhrase component.

^n^GVHD: graft-versus-host disease.

^o^Chemo: chemotherapy.

^p^DM: dermatomyositis.

^q^PG: yogerma gangrensoum.

^r^sweets: Sweet syndrome, also called acute febrile neutrophilic dermatosis.

^s^Clinicians could indicate other options using a free text box.

^t^gen: general.

^u^CC: continuity clinic.

^v^SDO: supportive dermatology-oncology.

#### Phase 2: Exploring Preintervention Pain Points and Adapting the Intervention

Semistructured interviews were conducted to further understand phase 1 pain points and inform early adaptations to the intervention. Clinicians and staff who had worked with the preintervention and postintervention workflows were invited via email (with 2 reminders) to participate in 30-minute phone interviews between April and May 2021. A total of 15 interviews were held by EASG or AA with 5 of 5 dermatologists, 5 of 5 residents, and 6 of 13 schedulers. Interviews were audio recorded and lasted for 30 to 60 minutes. Interviews informed both phases 2 and 3.

Data were analyzed, deductively and inductively, using a multiphase analysis approach that leveraged rapid analytic procedures to extract early themes, consensus coding of transcripts, and a matrix analysis [19]. In brief, EASG and AA summarized individual interview transcripts independently, reviewed summaries, had consensus discussions, and consolidated summaries into a matrix to identify themes and compare across interviewees. Identifiable information was removed from transcripts to maintain anonymity.

#### Phase 3: Evaluating the Intervention and Identifying Persisting Challenges

##### Overview

Mixed methods were used to evaluate the impact and sustainability of the intervention, the SmartPhrase and associated workflow. Specifically, qualitative interview data, scheduling data, and EHR messaging data were triangulated and consolidated and interpreted in parallel.

##### Perceptions of the Intervention’s Early Impact and Its Sustainability

The semistructured interviews explored interviewees’ perceptions of the early impact of the intervention on follow-up timeliness, workflow and workload, its potential sustainability, and persisting challenges for phase 3 (see phase 2 for methods and analysis).

##### Timeliness of Follow-up

The impact of the intervention on the timeliness of scheduling, completion of follow-up, and messaging workload was assessed by comparing two periods: (1) preimplementation (January 1 to February 28, 2021) and (2) postimplementation (April 1 to May 31, 2021). March 2021 was excluded, as the SmartPhrase was enabled on March 22, 2021. Data were extracted for all patients who received an inpatient dermatology consultation, were discharged from the hospital (ie, inpatient, observation, and emergency department [ED] encounters) within 1 of the 2 evaluation periods, and were expected to need an outpatient follow-up dermatology visit, that is, hospitalization had current procedural terminology codes indicating potential need for follow-up care (Multimedia Appendix 3). Follow-up visits scheduled and completed within 90 days of discharge were included; those scheduled or completed more than 90 days postdischarge were unlikely to be related to the hospitalization. Outcomes included (1) proportion of patients completing a follow-up visit within 90 days postdischarge, (2) proportion of patients completing a follow-up visit within 14 days postdischarge (postdischarge goal of department), and (3) days from inpatient discharge to completed follow-up. Descriptive statistics are reported. *P* values were calculated using chi-square tests for categorical outcomes and 2-tailed *t* tests for continuous outcomes.

##### Staff Messaging

EHR-based messaging volume data, specifically in-basket messaging in Epic [20], was used as a proxy for communication workload, as it was a commonly used and measurable. Sent messages were extracted for 5 inpatient dermatologists and 8 dermatology residents (2 per month) involved in inpatient care during the 2 periods. Of 13 scheduling staff, 12 schedulers sent messages during the preimplementation period and 11 schedulers during the postimplementation period. Messages related to scheduling patients who received an inpatient consultation and completed an outpatient follow-up visit within 90 days of discharge were identified using a keyword search (Multimedia Appendix 4). A total of 2 outcomes are reported for the two periods: (1) number of follow-up patients associated with staff messages and (2) number of in-basket messages sent.

### Ethics Approval, Informed Consent, and Participation

This project received a nonresearch determination from the Stanford University institutional review board (IRB-60382). Interviewees provided verbal informed consent before the initiating the interview and recording, and all responses were kept confidential and anonymous. Detailed interview notes were taken when participants declined to be recorded.

## Results

### Phase 2: Exploring Preintervention Pain Points and Refining the Intervention

#### Overview

**Figure 2:**
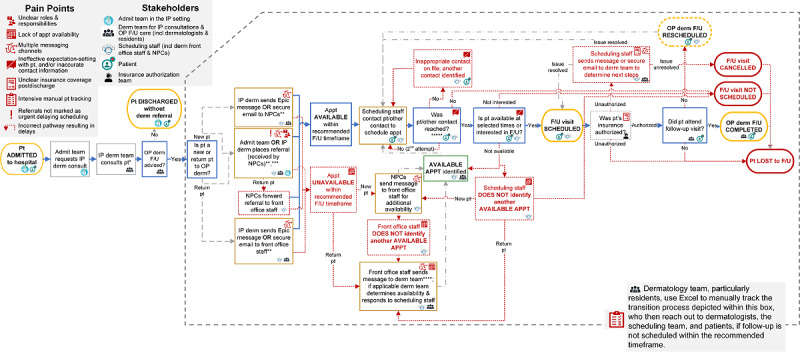
Expanded preintervention (ie, baseline) map informed by insights from scheduling and dermatology team through semistructured interviews in phase 2, exploring preintervention pain points and refining the intervention, of a quality improvement project aiming to improve timeliness of transitioning patients from inpatient-to-outpatient dermatology and reduce workload. *Inpatient dermatology consult completed in person or via e-consult. **Could occur pre or postdischarge. If before, scheduling team may wait to initiate scheduling until patient is discharged. *** If referral not marked as urgent, could result in a 1-2 week scheduling delay. ****Dermatology team, particularly residents, manually track patient transitions in Excel, who reached out to dermatologists, scheduling staff, and patients if follow-up not scheduled within the recommended timeframe. *****Patient contacted by phone up to two times and sent a mailed letter.Admit: Admitting; Appt: Appointment; Derm: dermatology; F/U: follow-up; incl: including; IP: inpatient; NPC: new patient coordinator; OP: outpatient; pt: patient.

Interviewees reported that the preintervention workflow had a high risk for communication errors, delays, losing patients to follow-up, and potentially adverse patient outcomes. Unclear roles and responsibilities, multiple messaging channels, limited patient input, and intensive manual tracking of patients were identified as local barriers. Lack of appointment availability and insurance authorization issues were important structural barriers. Supporting quotes are in Tables 2 and 3. Barriers were overlaid onto the original process map (Figure 2).

**Table 2:** Exemplary quotes from interviews with dermatologists, residents, and scheduling staff describing preintervention pain points related to the preintervention scheduling workflow for transitioning recently discharged patients to outpatient dermatology for follow-up care and associated workload lacking a standard process.

Theme: preintervention scheduling workflow lacked a standard process	Exemplary quotes
Overall perceptions	“...our gut feeling was that it didn’t work. We’re always so nervous that something falls through the cracks, and we don’t know about it” (dermatologist). “...before the SmartPhrase the system was just based on messaging and it was just a lot of work for everyone. I think that things fell through the cracks as well there” (resident). “...a lot of backend work that I just don’t think is that efficient, because it’s never actually solving the problem. It’s like, we’re just kind of patching things up, patient by patient, making sure one person doesn’t falls through the cracks. But we’re just not able to account for everybody that way” (dermatologist).
Unclear roles and responsibilities	“There’s so many residents, and we’re rotating so much, that you have to have a really well set up system that everyone’s involved in. Otherwise, things will for sure fall through the cracks” (resident). “...wasn’t honestly that clear whether it was the primary team that was sending the referral or whether we would send the referral [...] sometimes we were having the primary team send the referral. Then you would be reaching out to the medicine [admitting] team, like, ‘Hey, could you please put the referral?’ But I think just because I knew that I could send in the referral and it saves them a step, I would usually just do it anyway. I just felt like it was more work to have them do it. And I know they’re busy as well” (resident).
Multiple messaging channels	“...sometimes residents would message through Epic® and then sometimes it would be through email. So, it wasn’t always consistent where these messages ‘lived.’ And then sometimes the messaging would actually take place in the referral itself. So, there were just so many different places for us to keep track of” (dermatologist). “...I look at my schedule an unhealthy number of times every day” (dermatologist). “...they are all small tasks but then when you’re seeing two to four or five new patients a day, and then discharging a similar number, it adds up, doing those messages and referrals and trying to keep track” (resident). “I would send the referral to, and then would send a staff message as well, to the front office staff...patient was admitted for this reason, we might follow up. I will try to specify the timeframe and if there was a specific provider. But I obviously wasn’t always including every detail that they might need to help set the appointment” (resident). “...it’s very important that when creating the referral...that the appointment is marked urgent. That’s the most important thing to make sure that the patients are scheduled in a timely manner” (scheduler).
Limited patient and caregiver input	“...patients oftentimes have complex medical problems...the fact that they have a lot going on, it would be easy for them to not prioritize their skin issues” (resident). “...we need to ask the patient if they want an appointment because we actually were getting a bunch of patients scheduled, who then canceled same day or just no-showed, because they actually didn’t want follow-up. [...] it’s just very important to...engage the patient in that decision” (dermatologist).
Intensive patient tracking	“...we probably micromanage it. We won’t let go until something’s happened. So I think that feeling of worry that something is going to fall through the cracks led us to all universally micromanage more and send a lot of messages, want to see the confirmation if a patient doesn’t show then go back and ask, and then resend referrals again, and have schedulers call” (dermatologist). “...sometimes patients are anticipated to be discharged and then the resident will send the message to the staff, put in the referral and then the patient ends up having another reason for the stay in the hospital longer than what we anticipated. So by the time the schedulers call, the patients are still admitted in the hospital and so they’re not able to appropriately gauge when would be a reasonable time to schedule the patient” (resident).

**Table 3:** Exemplary quotes from interviews with dermatologists, residents, and scheduling staff describing preintervention pain points related to structural barriers for transitioning recently discharged patients to outpatient dermatology for follow-up care and associated workload.

Theme: structural barriers	Exemplary quotes
Limited appointment availability	“...they are all small tasks but then when you’re seeing two to four or five new patients a day, and then discharging a similar number, it adds up, doing those messages and referrals and trying to keep track” (resident). “...for every message that we send asking for a patient to be seen in follow-up within a couple of weeks, I think almost 100% of them, we then get a message back saying, ‘Please advise on where to schedule? There are no openings’” (dermatologist). “...one thing I’ve noticed that has been quite effective is, if the patient is going to be following with the attending that is seeing the patient, sometimes attendings pull up their calendar in the room and pick a date that day, and let the patient know. Then coordination becomes much more straightforward...” (resident).
Insurance authorizations	“...a patient can get scheduled for a visit regardless of their insurance status, whether or not they should go because they have to pay out of pocket is another story, but the lack of authorization doesn’t prevent scheduling teams from putting them on the schedule at a slot” (dermatologist). “...why can’t the process for insurance authorizations start when the patient’s still in the hospital? Why can’t we identify that we may encounter problems when they’re still in the hospital as opposed to waiting until they’re discharged?” (dermatologist).
Early implementation problems	“If they just mark it as urgent and then just put like hospital or ED discharge in the title, then we know that that’s a priority when we’re scanning, especially right now, the front desk we’re short. So, we’re not able to get to messages like we were before. So, marking it as urgent and just kind of giving us a heads up. It lets us know that those need to be a priority and worked on first” (scheduler). “...there’s a lot of lack of trust in the process. We’ve piloted different things in the past that stick around for a little bit, and then you’re back to normal again, so I always feel this sense of really needing to have ownership over it and closely monitoring to make sure it’s happening” (dermatologist).

#### Unstandardized Preintervention Workflow

##### Unclear Roles and Responsibilities

Residents felt responsible for care transitions, but they were unsure whether the admitting (ie, nondermatology) or consulting dermatology team was responsible for initiating a referral for outpatient follow-up (Table 2). Consequently, several residents found it easier to submit referrals themselves with a separate message to schedulers and consulting dermatologist. When urgent, some residents also called patients directly or sent additional staff messages to accelerate the process. However, residents rotate monthly, creating opportunities for inconsistencies in workflow and to lose patients during transitions. Residents also did not have role in outpatient dermatology during their 1-month inpatient dermatology rotation. In contrast, other academic dermatology programs have created discharge clinics where residents can provide follow-up care to achieve care continuity [21].

##### Multiple Messaging Channels

Scheduling relied heavily on back-and-forth messaging among interviewees through various channels, including referrals, in-basket messaging, and email (Table 2). Communication channels used depended on who initiated the referral, whether the patient was considered a new or return patient to the outpatient dermatology clinic, and whether the request was marked with “ASAP”; referrals not marked with “ASAP” were deprioritized with patient outreach occurring within 1 to 2 weeks, delaying care. Within this complex process (Figure 2), referrals were occasionally sent to the wrong staff members, and routinely lacked sufficient information were sent to schedule patients requiring further messaging among the team.

##### Limited Patient and Caregiver Input

Even with timely and adequate information, schedulers struggled to reach and schedule patients, as patients were unaware of the need or reason for follow-up care (Table 2). Clinicians recognized this may be especially challenging for complex patients juggling many medical issues. Engaging patients and caregivers in shared decision-making predischarge and gauging their interest or ability to attend a follow-up appointment was considered necessary to accommodate patients, improve response to schedulers’ phone calls, and decrease the number of patients who decline or miss follow-up. This may be particularly challenging in the context of consultative dermatology. Although the inpatient dermatology team closely followed most patients during their hospitalization, there was high variability exposure to each patient and their caregivers. There may be variability in the prioritization of dermatological conditions and follow-up care depending on other health conditions and their admitting care team.

##### Intensive Patient Tracking

Scheduling and dermatology team lacked closed-loop communication; the dermatology team rarely knew whether patients were scheduled for follow-up, leading to persistent worry about losing patients (Table 2). Primarily residents, but also dermatologists, manually kept lists of patients discharged to monitor scheduling activities and follow-up status. This required repeatedly checking the EHR and messaging other scheduling and dermatology team members. Discharge delays further disrupted scheduling of follow-up, but residents only knew of these delays through this tracking and they “probably micromanage[d] it” (dermatologist).

#### Structural Barriers

##### Limited Appointment Availability

The lack of appointment availability within the desired time frame also contributed to additional messaging (Table 3). When suitable timeslots were not available, extra messages were sent between scheduling staff and clinicians to find additional availability (Figure 2). All interviews considered this process burdensome. Timeliness of follow-up care was considered more important than ensuring continuity of care, but some schedulers and residents believed scheduling with the consulting dermatologist is easier, as the dermatologist could suggest specific timeslots or allow overbooking. The limited appointment availability is particularly challenging for high-volume specialties, including dermatology, that receive referrals from a variety of sources with various urgency.

##### Insurance Authorizations

According to the dermatology team, “...a patient can get scheduled for a visit regardless of their insurance status” (dermatologist), but insurance authorization could disrupt and delay follow-up plans (Table 3). Thus, obtaining approval before discharge could facilitate appropriate follow-up care plan.

#### Early Implementation Problems and Resulting Intervention Adaptations

In the first weeks of implementation, intervention vulnerabilities included unforeseen staffing shortages and referrals not marked as “ASAP,” a key step in submitting the referral with the SmartPhrase (Table 3). These contributed to scheduling delays and one anecdotally reported readmission. Consequently, manual tracking of patients continued as the dermatology team was uncertain whether the new workflow worked as intended.

**Figure 3:**
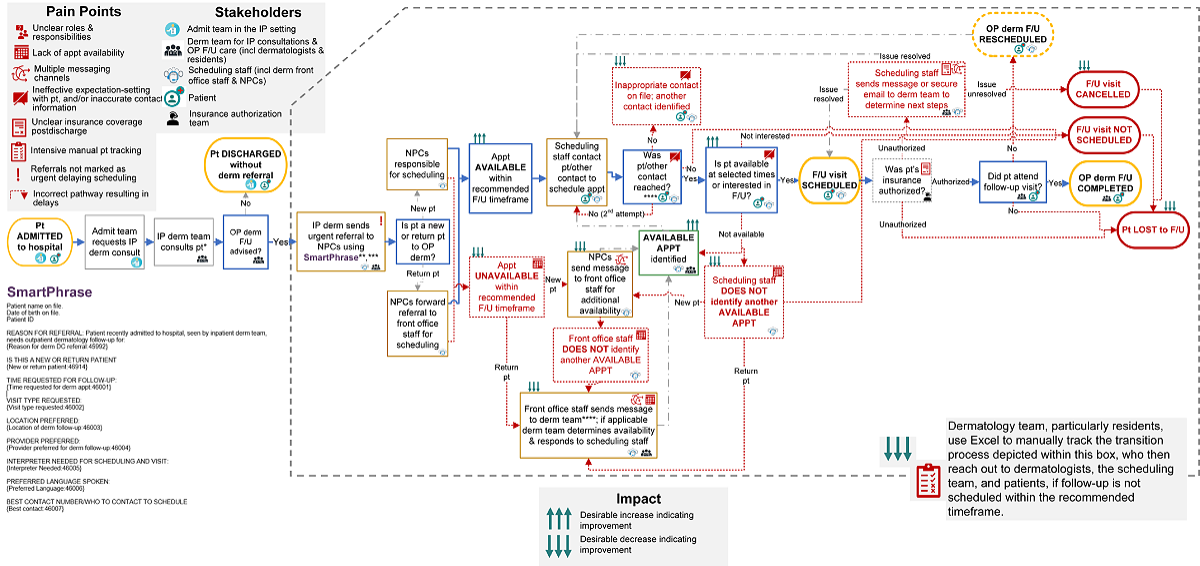
Process map for intervention, a SmartPhrase and associated workflow, developed using insights from schedulers, dermatologists, and residents through semistructured interviews, scheduling data, and messaging data. Data were collected in phase 2, exploring preintervention pain points and refining the intervention, and phase 3, evaluating the intervention and persisting challenges, of a quality improvement project aiming to improve timeliness of transitioning patients from inpatient-to-outpatient dermatology and reduce workload. *Inpatient dermatology consult completed in person or via e-consult. **Could occur pre or postdischarge. If before, scheduling team may wait to initiate scheduling until patient is discharged. *** If referral not marked as urgent, could result in a 1-2 week scheduling delay. ****Dermatology team, particularly residents, manually track patient transitions in Excel, who reached out to dermatologists, scheduling staff, and patients if follow-up not scheduled within the recommended timeframe. *****Patient contacted by phone up to two times and sent a mailed letter.Admit: Admitting; Appt: Appointment; Derm: dermatology; F/U: follow-up; incl: including; IP: inpatient; NPC: new patient coordinator; OP: outpatient; pt: patient.

Within the first month, early inconsistencies were addressed by onboarding and training the weekend and overnight dermatology residents on the intervention. Early on, the SmartPhrase was being used inconsistently because of failure to update weekend residents and overnight residents of the new workflow (Table 3). Furthermore, referrals were not being marked as “ASAP,” which was identified as a crucial step to ensure the referral was processed urgently. As a response, the SmartPhrase was edited to include text that emphasized that all inpatient referrals need to be marked “ASAP” (Table 1). This information was also included in monthly email reminders to all residents rotating onto the inpatient service (as well as those covering weeknights and on weekends), inpatient handbook for clinicians and residents, verbal sign-out by residents, and yearly introductory presentation for residents. Table 1 presents the final SmartPhrase, and Figure 3 displays the workflow.

### Phase 3: Evaluating the Intervention and Persisting Challenges

#### Overview

**Figure 4:**
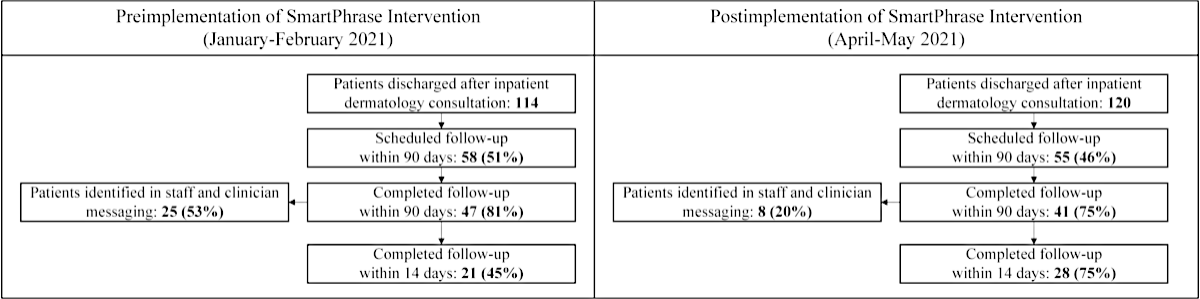
Number (percentage) of patients who received inpatient dermatology consultation and potentially needed were scheduled for and completed their outpatient postdischarge follow-up and number (percentage) of patients identified in staff and clinician messages related to inpatient-to-outpatient care transitions before and after implementation of an intervention are reported. The intervention, a SmartPhrase and associated workflow, was implemented as part of a quality improvement project aiming to improve timeliness of transitioning patients from inpatient-to-outpatient dermatology and reduce workload.

During the pre- and postimplementation periods, 114 and 120 patients, respectively, received an inpatient consultation from the dermatology team, were discharged from the hospital, and potentially needed follow-up (Figure 4). Qualitative themes (supporting quotes in Table 4) and quantitative data were triangulated and are presented in subsequent sections.

**Table 4:** Exemplary quotes from interviews with dermatologists, residents, and scheduling staff perceptions of intervention to improve the timeliness of transitioning recently discharged patients to outpatient dermatology for follow-up care and associated workload.

Theme: evaluating the intervention	Exemplary quotes
Timeliness of scheduling and follow-up	“...just one system, one process that we now have. So, instead of multiple emails being sent, staff messages being sent, there is just one unified way to do it. So, I think that makes it much easier for residents” (dermatologist).
Messaging workload	“...there’s just more information that’s built into the SmartPhrase right off the bat, so there’s less need for messaging back and forth between the schedulers, residents, faculty members and nursing staff” (dermatologist). “...the SmartPhrase does decrease workload in terms of hours, our time spent on documentation and administrative tasks. [...] one of the biggest areas that can lead to burnout is the documentation burden, so I think SmartPhrases definitely help with that” (resident). “[Before the introduction of the discharge SmartPhrase] closing the loop did not happen. I think that’s the biggest change is making the physicians aware of when the patient will be scheduled and that they are on the schedule” (scheduler).
Clinical burden	“...for the handful that I’ve dealt with this week that needed follow up, I still feel like I’ve been pretty involved in making sure they’re on the schedule” (dermatologist). “There’s just more time and mental energy to spend on urgent items, clinical direct care...but also the exhaustion of worry about going back...that finally took me out of my worry sphere. [...] the ability to not worry about that has been tremendously helpful because that even goes beyond the physical time we spend in the chart...that constant worry in the back of your head that maybe it’s still not done has been eliminated” (dermatologist). “I don’t really see a big difference as far as the number of staff messages. I just think the efficiency and communication is better with the SmartPhrase and closing the loop, because once you send out the message to the referring physician and the doctor that you’re scheduling with, that’s just where it ends” (scheduler). “Maybe a little bit improved [my wellbeing], but very minimally. [...] it’s just a small fraction of our workload as a consult resident. That it just doesn’t proportionally have that much effect” (resident).
Integrating patient and caregiver input	“They’re [patients] involved from the beginning, we usually have it at bedside to get their preferences. And then part of the discharge checklist is we clarify their preference for care, whether they want it to be in person or video, sort of a timeline, the preferred contact method...” (resident). “...it’s very helpful when you put [in the SmartPhrase]...the best person to contact for the patient because sometimes it’s not the patient. Sometimes it’s the patient’s husband. It’s the daughter. It’s the long-term rehabilitation facility, so that makes it really helpful for us to know who’s the primary person to contact to get the patient scheduled” (scheduler).

#### Timeliness of Scheduling and Follow-up

The intervention was well-accepted by all interviewees (Table 4). The dermatology and scheduling teams were familiar with the SmartPhrase feature, as it has been used to create other templates used in their daily practice. The intervention was reported to be easily adopted and facilitated efficient scheduling of discharged patients for outpatient follow-up. However, the intervention did not substantially impact the proportion of patients with scheduled or completed follow-ups. The proportion of patients scheduled for a follow-up visit with a 90-day postdischarge period did not improve, 50.9% (58/114) preimplementation period versus 45.8% (55/120) postimplementation period (*P*=.44; Figure 4), nor did the proportion of patients with completed follow-up visits within the same timeline (47/58, 81% vs 41/55, 75%, respectively; *P*=.41). The overall time from hospital discharge to follow-up completion decreased slightly, but not significantly, from before the implementation to after the implementation (mean 20.4, SD 19.3 to mean 17.8, SD 20.8 days; *P*=.55). However, the proportion that completed their follow-up visit within 14 days of discharge significantly increased from 45% (21/47) to 68% (28/41), before the implementation to after the implementation, respectively (*P*=.03).

#### Messaging Workload

**Figure 5:**
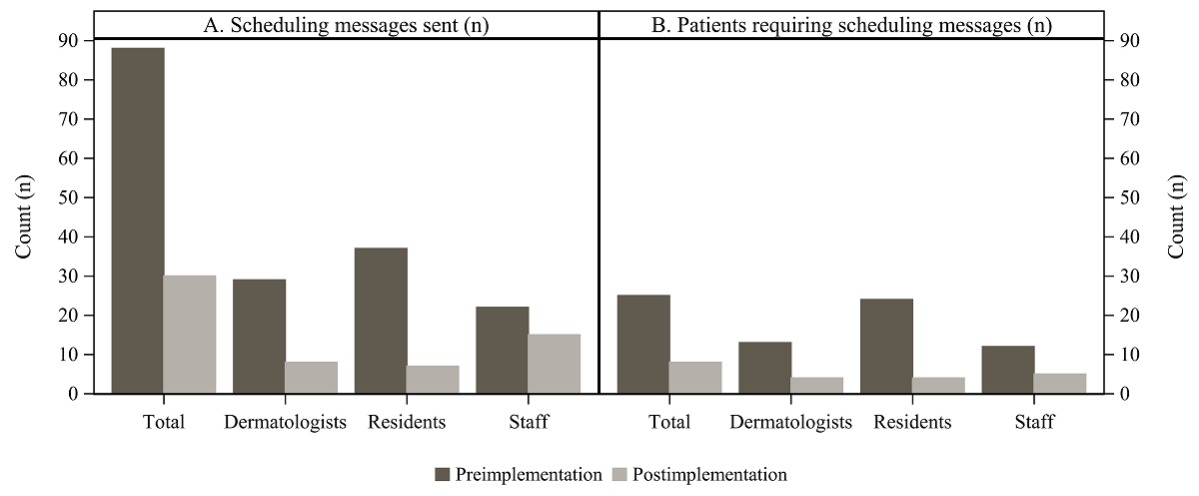
(A) Total number of messages sent by dermatologists, residents, and staff per patient and (B) total number of patients who completed a follow-up visit within 90 days of discharge and required messaging to schedule their visit and prior pre- and postimplementation of the intervention, a SmartPhrase and associated workflow, for a quality improvement project aiming to improve timeliness of transitioning patients from inpatient-to-outpatient dermatology and reduce workload.

The volume of messages related to care transitions decreased after intervention; dermatology and scheduling teams sent a total of 88 messages before the implementation and 27 messages after the implementation (Figure 5A). The group sending the most messages also shifted; before the implementation, almost half of the messages were sent by residents, whereas in the postimplementation period, the majority of the messages were sent by schedulers (Figure 5A). Furthermore, messages were also sent for fewer patients; of the patients who completed follow-up within 90 days of discharge, 53% (25/47) of patients were associated with messages before the implementation and 20% (8/41) of patients were associated with messages after the implementation (Figure 5B). This aligned with interviewee perceptions that completed SmartPhrases provided the schedulers with sufficient information to schedule a follow-up and reduced back-and-forth messaging and time spent in the EHR (Table 4).

#### Clinical Burden

Perceived impact of the intervention on clinical burden was mixed among dermatology and scheduling staff (Table 4). The standardized workflow, reduced messaging, and more consistently closing the communication loop when a follow-up was scheduled were perceived to reduce burden. Most dermatologists and residents reported that the intervention allowed them to shift their focus onto more pressing needs and brought a sense of relief and improved well-being. However, 1 dermatologist and 1 resident did not note differences in workload because of additional back-and-forth messaging during early implementation.

#### Integrating Patient and Caregiver Input

The intervention prompted the inpatient dermatology team to engage patients and caregivers in bedside shared decision-making before discharge to obtain necessary information for scheduling. Interviewees reported that this resulted in more accurate and detailed information that facilitated scheduling and minimized delays (Table 4). Clinicians were also prompted to discuss the importance and purpose of follow-up with patients and caregivers, which was helpful to schedulers as “...patients are informed about the referrals...so they expect us to call them” (scheduler).

#### Sustainability and Persistent Challenges

The perceived and actual benefits of this easy-to-use intervention (Figure 3) led all interviewees to believe that it was sustainable (supporting quotes in Multimedia Appendix 5). However, interviewees reported persisting challenges: (1) inconsistent timing of when to initiate scheduling effort (ie, before or after discharge); (2) lack of process for tracking patients with missed follow-ups; (3) follow-up reason not always documented in the SmartPhrase (Table 1); (4) lack of systematic training for new residents and scheduling staff, roles with frequent turnover, and compromising trust; (5) lack of a dedicated coordinator to own and manage care transitions; and (6) hesitation to fully trusting the intervention. The best timing of scheduling activities is dependent on patient discharge from inpatient settings, but the inpatient dermatology team was not always involved in discharge decisions nor notified about delays. Structural barriers also remained, including continued lack of appointment availability within the recommended follow-up timeline and postdischarge insurance denials leading to cancelations and delays.

## Discussion

### Principal Findings

Transitioning patients from inpatient consultation services to outpatient dermatology for follow-up is a complex process. Dermatology and scheduling teams reported undue burden owing to several pain points: lack of standardized workflow; limited patient and caregiver involvement in predischarge planning; and burdensome, manual tracking of patients through their transition. Patients were generally satisfied with the transition process but identified persisting issues around communication and expectation setting during discharge planning and care coordination and prioritization, especially for medically complex patients. Identified issues and pain points were partially addressed by the intervention, a SmartPhrase and associated workflow, by prompting the inpatient dermatology team to collect information needed for scheduling at bedside and standardizing the communication between the dermatology and scheduling teams. The intervention was widely accepted, was easy to use, reduced the workload, and increased the proportion of patients receiving follow-up within the desired 14-day postdischarge timeline. Fewer patient transitions required EHR scheduling–related messaging, and messaging workload shifted from residents to the scheduling team. Although the intervention was viewed as sustainable, local and system-level challenges to effective care transitions remain.

### Comparisons With Previous Literature and Implications

Burnout among clinicians and health care workers has been identified as a consequence of intensive EHR use, including documentation, inbox messaging, and other tasks that increase mental load and time spent caring for patients [22-24]. These activities were plentiful in the preintervention workflow at the present organization, which contributed to stress and worry among team members. The flexibility and accessibility of the SmartPhrase feature in the Epic EHR allowed rapid development of a stakeholder-informed template that consolidated patient information needed for scheduling follow-ups into a standard referral. Almost all clinicians, residents, and scheduling staff reported at least some improvement in their workload, stress, and well-being after implementation, which aligned with the decrease in messaging seen after the implementation. Other studies have also found that when strategically used, the SmartPhrase is easy to use and a rapidly deployable solution for projects with short timelines and limited resources needing to consolidate documentation, streamline communication, and decrease workload [25,26].

Providing patients with timely access to follow-up care after hospitalization has many documented benefits to the patient and health care system [27-31]. Research has shown that faster postdischarge follow-up may prevent readmissions and mortality in irritable bowel syndrome, heart failure, and Hospital Readmissions Reduction Program’s priority conditions, such as acute myocardial infarction and pneumonia [27-30]. Although this QI project did not increase the proportion of patients receiving follow-up or average time between discharge and follow-up visit, the proportion of patients receiving dermatology follow-up care within the desired 14-day postdischarge timeline was 68% after the intervention. This is similar to a reported proportion of patients accessing any ambulatory follow-up 14 days after hospitalization (50%-67%) [32-34] and 30 days after an ED visit (71%) [35] related to a variety of concerns and greater than the proportion of patients with heart failure seeking follow-up care 30 days after the ED visit (23%) [36]. This suggests that the intervention addressed preintervention concerns around prioritization or deprioritization and (lack of) awareness of dermatological issues, which are important for adherence to care plans, including follow-up visits [35,37]. However, gaps remain in the coordination of postdischarge dermatological care. Baseline clinical factors and social risk factors [29,31,38] have been shown to be related to follow-up attendance and benefits but were not explored here because of the small sample size. We were also unable to explore if follow-up care completion or timing was impacted by a patient’s specific dermatological diagnosis, and dermatology conditions vary widely in urgency, timeline of treatment, and thus appropriate timing of follow-up. Further investigation is needed to understand how to tailor follow-up recommendations to patient factors and dermatologic diagnosis and to develop patient-centered workflows that promote appropriate and timely postdischarge care.

Challenges in care transitions persist, but this mixed methods evaluation enabled the identification of the next steps for improvement [2,7,39-42]. In particular, it was recommended that the inpatient dermatology team should obtain additional information, such as best time to call and follow-up purpose, at bedside and clarifying the workflow for when discharge is delayed or when a patient cancels, reschedules, or misses their follow-up. Offering teledermatology could also help patients receive timely follow-up care [17]. Systematic onboarding of new clinicians, residents, and staff members are also needed to sustain the intervention. The current intervention heavily relies on team members in roles with high turnover and frequent shifts in roles or responsibilities, specifically residents and scheduling staff. Other research suggests that a dedicated owner of the process, such as a care coordination team [42,43] or discharge clinic [21], is effective. However, these are resource-intensive solutions and may not resolve the pervasive problem of lack of appointment availability. Dedicated timeslots allotted for discharged patients in each clinician’s schedule or a “discharge clinic” may somewhat help [21]. However, interviewees worried that they would give patients less flexibility. Further exploration of such interventions is warranted.

### Limitations

There are several limitations to this evaluation. First, this single-center study may not be generalizable, although some of the identified pain points, such as poorly defined roles and responsibilities and nonstandard communication channels, have been previously reported [39-42]. Second, we were unable to accurately identify all patients needing follow-up care and SmartPhrase use, as these were not documented in unique, extractable data fields during this study. Thus, the denominator for the study is not precise, but there is no reason to expect that there was systematic difference in identification of patients with current procedural terminology codes between the 2 periods. These issues have been resolved by the institution’s EHR team since the completion of this study. Third, only in-basket messaging data were available, which were the most common, but not the only, channel for communication (eg, email, phone, and instant messaging). Finally, because of limited resources, we were unable to capture patient perspectives during implementation.

### Conclusions

A well-accepted, easy-to-use intervention, the SmartPhrase and associated workflow, improved the proportion of patients receiving follow-up dermatology care within 14 days of discharge but did not impact the proportion of patients scheduled or completing follow-up within 90 days of discharge. It also facilitated efficient scheduling of discharged patients with substantial reduction in staff messaging, alleviating the scheduling burden; clinicians, residents, and scheduling staff reported less stress and improved well-being. The SmartPhrase can be adjusted based on user experience, making it flexible for long-term sustainability. The multipronged approach to evaluate this intervention not only informed the QI project but also provided a foundation for future efforts, which will be applied to address the remaining challenges around care transitions. We found that a simple stakeholder-informed solution can be created and implemented quickly with a standard EHR figure that results in a positive impact; this approach could be easily applied to care transitions beyond dermatology.

## Appendix

Multimedia Appendix 1 Description of qualitative methodology and participating patients and caregivers that elucidated experiences with inpatient-to-outpatient dermatology care transitions.

Multimedia Appendix 2 Findings and exemplar quotes from patient (n=14) and caregiver (n=1) interviews organized by theme.

Multimedia Appendix 3 Current procedural terminology (CPT) codes that were associated with inpatient dermatology consult and provider that were used to identify patients who had an inpatient consult with dermatology and who may need follow-up care in outpatient dermatology. Inpatient dermatology consultations were identified with the following CPT codes for both in-person consults and e-consults (primarily offered during pandemic) linked to a dermatologist during an inpatient encounter. As clinician recommendation on follow-up need or timeline could not be reliably extracted from the electronic health record, it was assumed that patients who received an inpatient dermatology consultation associated with these CPT codes may have needed follow-up care.

Multimedia Appendix 4 Keywords used to identify clinician and staff sent messages related to transitioning patients from inpatient care to outpatient follow-up care in dermatology.

Multimedia Appendix 5 Exemplary quotes from interviews with dermatologists, residents, and scheduling staff describing persisting challenges postimplementation of a SmartPhrase-enabled workflow to improve timeliness of patient transitions from inpatient-to-outpatient dermatology care and associated messaging workload.

## Abbreviations

ASAP: as soon as possible
ED: emergency department
EHR: electronic health record
NPC: new patient coordinator
QI: quality improvement

## Data Availability

The data that support these findings are available from the corresponding author on reasonable request.
