# Supplementary material for: Evolution of a Project to Improve Inpatient-to-Outpatient Dermatology Care Transitions: Mixed Methods Evaluation
Source: JMIR Dermatol. 2023 May 25;6:e43389. doi: 10.2196/43389 (PMC10335331; doi:10.2196/43389)
Supplement: Multimedia Appendix 1 [file derma_v6i1e43389_app1.pdf]

Multimedia Appendix 1. Description of qualitative methodology and participating patients and caregivers that elucidated experiences with inpatient-to-outpatient dermatology care transitions.

Qualitative Methodology

A stratified random sample of English-speaking patients/caregivers discharged from inpatient dermatology consultative service and referred to outpatient dermatology November 2020 to January 2021, were invited to semi-structured phone interviews. Of 59 eligible patients, 14 patients and 1 caregiver were interviewed February to March 2021 (Table 1). Transcripts were analyzed inductively by two qualitative experts and a dermatologist, leveraging rapid analytic procedures and inductive thematic analysis, with regular discussion to achieve reporting consensus.

Patient and Caregiver Characteristics

Patient demographic characteristics and type of outpatient follow-up visit (n=15)	
**Patient characteristics**	**Frequency, n**
**Age (years)**	
30-49	5
50-64	6
≥65	4
**Gender**	
Man	9
Woman	6
**Ethnicity**[*]	
Non-Hispanic/Non-Latino	9
Hispanic/Latino	3
**Insurance type**	
Private[†]	6
Public[‡]	9
**Dermatology subspecialty**	
Supportive dermato-oncology[**]	10
General dermatology	5
**Follow-up status**	
Complete (on-time)[§]	8
Complete (delayed)[¶]	3
Incomplete	4
**Follow-up visit type**[#]	
Video visit	9
In-person	2

NOTE. [*]As reported in the patient's electronic medical record; [†]Includes EPO, PPO and HMO insurance types; [‡]Includes Medicare and Medi-Cal; [**]refers to a collaboration between multiple oncology specialty clinics and *dermatology. It caters for oncology patients who experience dermatological complications as a result of cancer therapy;* [§]Follow-up appointments completed within the recommended timeframe; [¶]Follow-up appointments not completed within the recommended timeframe; [#]Restricted to patients with a complete follow-up visit
