# Supplementary material for: Evolution of a Project to Improve Inpatient-to-Outpatient Dermatology Care Transitions: Mixed Methods Evaluation
Source: JMIR Dermatol. 2023 May 25;6:e43389. doi: 10.2196/43389 (PMC10335331; doi:10.2196/43389)
Supplement: Multimedia Appendix 2 [file derma_v6i1e43389_app2.pdf]

Multimedia Appendix 2. Findings and exemplar quotes from patient (n=14) and caregiver (n=1) interviews organized by theme

Findings and exemplar quotes from patient (n=14) and caregiver (n=1) interviews organized by theme

Theme	Sub-theme	Sub-theme description	Exemplar quote
**Patient-physician communication and expectation-setting during discharge planning**	Quality of patient-physician communication and expectation-setting during discharge planning	Most interviewees were satisfied with their discharge plan. Several dissenting patients remarked that their expectations were met, although there were occasional evident mismatched expectations regarding treatment and necessity for dermatology follow-up. A few patients, particularly those who experienced serious (non-dermatological) medical events whilst at hospital, had limited recall of their interaction with dermatology, therefore the necessity of follow-up was sometimes unclear.	"…I think they continued to follow me maybe even longer than they needed to" (Pt11, 70-year-old male patient)
	Involvement of caregivers for successful care transitions	Patients' need for support from family or friends varied. Whilst most coped with their follow-up needs alone, some required scheduling and follow-up treatment support. A thorough explanation by the dermatology team at discharge was perceived useful for patients to relay information to caregivers.	"My wife did it [scheduled follow-up appointments] because I was just too out of it" (Pt4, 79-year-old male patient)
**Access to follow-up care: involvement of the dermatology team and care delivery via video**	Timeliness of follow-up care scheduling and availability of the dermatology team	Patients were generally very satisfied with their follow-up coordination experience. Patients for whom the dermatology team was heavily involved in coordinating follow-up care appreciated it, particularly if they experienced discharge delays or if coordination across multiple specialties was required. Ultimately, patients were pleased with the time from discharge to follow-up, even if they encountered delays, possibly because several perceived their dermatological issue as non-urgent.	"…the dermatology problem was not my main priority… I really felt fine with the time between discharge and my appointment." (Pt13, 63-year-old female patient)
	Patient's technological capacity and interest in follow-up video visits	Although not all patients were followed up via video, they agreed that video visits are convenient and easy, and provide better access to patients, particularly those who are immunosuppressed or reside far from the clinic. Almost all expressed future interest in using video visits with dermatology, particularly for minor issues and if considered clinically appropriate by the physician. Concerns raised were connectivity issues and ensuring adequate quality of photos sent pre-visit.	"…I'm immunocompromised ... there's no way I could go back to a clinic setting right now, so that was great. Plus, I didn't have to get in the car and drive all the way there. (Pt15, 66-year-old female patient)
**The medically complex patient: care coordination and prioritization across multidisciplinary teams**	Prioritization of non-dermatological issues	For several patients admitted for non-dermatological issues, prioritization and urgency of their dermatological care varied. Notably, four patients with complex medical issues perceived their dermatological problem to be less urgent and less concerning than their primary medical concern, albeit related to their primary medical issue. In fact, three of these patients declined dermatology follow-up, one of whom was uninsured and unemployed, forcing them to prioritize their most critical needs. Despite this, all these patients reported that their skin condition was improving with appropriate management of their main health concern.	"...I was more worried about the heart issue... the dermatology stuff, the legs were related to that heart issue. Once I was working more on that, the other issue was going away [...] I have to focus on the heart stuff right now." (Pt8, 55-year-old male patient)
	Coordinating care across multidisciplinary teams	Some medically complex patients reported that their primary provider (e.g., their oncologist) managed their holistic care. These patients followed-up with their main provider and postponed/ declined their dermatology follow-up appointment. A few SDO* patients mentioned that they visited hospital regularly and would appreciate coordinating dermatology follow-ups with those visits.	"…it's better if they [dermatology] can see me in the hospital. …because I am already there. [...] It's easier." (Pt10, 33-year-old female patient)
**NOTE.** * SDO – supportive dermato-oncology, refers to a collaboration between multiple oncology specialty clinics and *dermatology*			
