# Supplementary material for: Evolution of a Project to Improve Inpatient-to-Outpatient Dermatology Care Transitions: Mixed Methods Evaluation
Source: JMIR Dermatol. 2023 May 25;6:e43389. doi: 10.2196/43389 (PMC10335331; doi:10.2196/43389)
Supplement: Multimedia Appendix 3 [file derma_v6i1e43389_app3.pdf]

Multimedia Appendix 3. CPT codes that were associated with inpatient dermatology consult and provider that were used to identify patients who had an inpatient consult with dermatology and who may need follow-up care in outpatient dermatology. Inpatient dermatology consultations were identified with the following CPT codes for both in-person consults and e-consults (primarily offered during pandemic) linked to a dermatologist during an inpatient encounter. Since clinician recommendation on follow-up need or timeline could not be reliably extracted from the EHR, it was assumed that patients who received an inpatient dermatology consultation associated with these CPT codes may have needed follow-up care.

Type of Consult	CPT Codes
e-consult	99446, 99447, 99448, 99449, 99451
in-person	99251, 99252, 99253, 99254, 99255, 99221, 99222, 99223, 99224, 99225
