# Supplementary material for: Evolution of a Project to Improve Inpatient-to-Outpatient Dermatology Care Transitions: Mixed Methods Evaluation
Source: JMIR Dermatol. 2023 May 25;6:e43389. doi: 10.2196/43389 (PMC10335331; doi:10.2196/43389)
Supplement: Multimedia Appendix 4 [file derma_v6i1e43389_app4.pdf]

**Multimedia Appendix 4.** Key words used to identify clinician and staff sent messages related to transitioning patients from inpatient care to outpatient, follow-up care in dermatology

| | |
|---|---|
| discharge | new visit |
| dc (abbreviation for discharge) | npv (abbreviation for new patient visit) |
| d/c (abbreviation for discharge) | rpv (abbreviation for return patient visit) |
| follow up | outpatient |
| follow-up | rpv (abbreviation for return patient visit) |
| followup | reschedule |
| inpatient | referral |
| inpt (abbreviation for inpatient) | schedule |
| new patient | |
