# Supplementary material for: Evolution of a Project to Improve Inpatient-to-Outpatient Dermatology Care Transitions: Mixed Methods Evaluation
Source: JMIR Dermatol. 2023 May 25;6:e43389. doi: 10.2196/43389 (PMC10335331; doi:10.2196/43389)
Supplement: Multimedia Appendix 5 [file derma_v6i1e43389_app5.pdf]

Multimedia Appendix 5. Exemplary quotes from interviews with dermatologists, residents, and scheduling staff describing persisting challenges post-implementation of a SmartPhrase-enabled workflow to improve timeliness of patient transitions from inpatient to outpatient dermatology care and associated messaging workload

**Persistent Challenges**	**Exemplary Quotes**
**Post-intervention Lack of a Standard Process**	
Inconsistent timing of Scheduling Activities	"...having the ability to take care of scheduling while the patient's still in the hospital ... There's less chance for them falling through the cracks if we actually get everything done before they go." (Dermatologist)
	"…I've experienced a couple of patients that are still in the hospital, and they usually don't like to schedule because they're not discharged or they're not feeling well. Also, the ones that I have scheduled, while they were in there, they're discharged, something happens, and then they're not discharged and the patient misses the appointment, and we're not notified that the patient no showed because they're still inpatient." (Scheduler)
	"...a lot of times we'll put in the referral a few days before we think they're going to be discharged rather than last minute, especially people who are sicker, who are going to require very close outpatient follow-up. We'll often put it in sometimes even a week before they're going to leave the hospital. [...] a problem that we have encountered is, when we'll do that, if the schedulers call the patient or sometimes we'll even call, they'll just look in the chart and they'll see, oh, the patient is still in inpatient. So they defer scheduling the follow-up when in reality, they could still try to call the patient's cell phone that they have with them in the hospital to make the appointment so that it doesn't fall through the cracks. I think that would be one area to improve." (Resident)
Patient Tracking for Missed Follow-ups	"...if they [patients] no show, they just don't show up. ...we don't have a process to make sure that the discharge patients are checked in and ... follow through. Once we schedule the appointment, we just kind of move on to the next. Just because we schedule so many patients daily, there's no process for that." (Scheduler)
Lack of Patient Understanding of Follow-up Importance	"[including a brief description of what the patient was seen for] would help because I noticed sometimes when we call... for example the patient was like, 'Oh what am I coming in for again?' And it'd be nice to have just that background of knowing what they're coming in for and what we could tell them. [...]" (Scheduler)
Onboarding New Staff	"...hoping that this will be integrated into our discharge process, and that it's going to be widely used by all of the residents, faculty and schedulers, and that there will be a system in place to train or onboard new faculty, schedulers, and new patient coordinators." (Dermatologist)
Need for Dedicated Staff	"In an ideal world, I think having a staff member that was dedicated to checking the list and calling up to make sure that all the patients were seen would be great. But there's a fair amount of time and because the residents are constantly changing, having a consistent person that was checking on that would be helpful." (Resident)
**Pre-intervention Structural Barriers**[1]	
Continued Lack of Appointment Availability	"...the issue is always trying to just get the patient in for an appointment within two weeks or four weeks. I think that's mainly the issue with all this […] Even with the SmartPhrases, I feel like it's still difficult to get patients in within the desired time that the physician would say to get them." (Scheduler)
	"...we've always talked about setting up a very specific clinic for outpatient follow-up, so we're not trying to slot people in somewhere, but every Friday there's an inpatient follow-up clinic. And then that way it's just a dedicated clinic where these patients can be seen." (Resident)
	"…the problem with the discharge clinic is that it just is so dependent on someone's schedule [...] If we're going to think about discharge slots, they need to be a little bit more widespread. No one's in clinic five days a week, so they have to be spread out amongst people in a bit more of the holistic way." (Dermatologist)

[1]Insurance authorization process was identified as a barrier pre-intervention and remained a barrier post-intervention.
